# Physician-Modified TEVAR versus Hybrid Repair of the Proximal Descending Thoracic Aorta

**DOI:** 10.3390/jcm11123455

**Published:** 2022-06-16

**Authors:** Miroslav Dimitrov Yordanov, Alexander Oberhuber, Abdulhakim Ibrahim

**Affiliations:** Department of Vascular and Endovascular Surgery, University Hospital Muenster, 48149 Muenster, Germany; alexander.oberhuber@ukmuenster.de (A.O.); abdulhakim.ibrahim@ukmuenster.de (A.I.)

**Keywords:** physician-modified TEVAR (pmTEVAR), hybrid TEVAR (hdTEVAR), left subclavian artery (LSCA), type B aortic dissection (TBAD), thoracic aortic aneurysm (TAA)

## Abstract

There are different surgical options for the treatment of proximal lesions of the descending thoracic aorta. The aim of this study was to compare the outcome of physician-modified TEVAR (pmTEVAR) vs. hybrid repair of the thoracic aorta in terms of TEVAR with carotid-subclavian bypass (hdTEVAR). This was a single-centre, retrospective comparative study of all patients who underwent pmTEVAR and hybrid repair of the proximal descending aorta from January 2018 to June 2021. Primary outcomes were technical success, 30-day mortality, perioperative stroke, 30-day reinterventions and supraaortic access related complications. Secondary outcomes were patient survival, late complications, late reinterventions, and bypass/bridging stent patency. A total of 181 patients underwent TEVAR within the period of 42 months. In our study, only patients with proximal landing in zone 2 (*n* = 39) were included. A total of 5 of 15 pmTEVAR and 8 of 24 hybrid repair operations (33% vs. 33%, respectively) were performed due to aneurysms. Among the rest of the patients, 10 of 15 pmTEVAR and 16 of 24 hybrid operations (67% vs. 67%) were performed due to aortic dissection. Technical success was achieved in 100% of the patients. No significant difference in terms of postoperative complications could be detected in the early and midterm follow up period. The 30-day mortality was 12.5% in the hybrid repair group (*n* = 3) vs. 6.66% (*n* = 1) in the pmTEVAR group (*p* = 0.498). These patients underwent the operation in an emergency setting. No patient died after an elective operation. The causes of early mortality were major stroke (*n* = 2), haemorrhagic shock (*n* = 1) in the hybrid group and progredient spinal cord ischemia with tetraplegia and acute respiratory insufficiency (*n* = 1) in the pmTEVAR group. In conclusion, both therapies are robust techniques, with comparable patency rate and perioperative complications. pmTEVAR appears to be advantageous in terms of operation time and tendency to lower mortality rates.

## 1. Introduction

The standard open repair of the proximal descending thoracic aorta is nowadays elective, and predominantly for patients with connective aortic diseases. Despite the improvements in recent decades, open operations are still associated with a long recovery period, as well as relevant postoperative complications [1,2]. As a result, in recent years the Thoracic Endovascular Aortic Repair (TEVAR) has become the first-line therapy for patients with type B aortic dissection (TBAD) or complex thoracic aortic aneurysms (TAA) [3]. However, when the aortic lesion is close distal to the left subclavian artery (LSCA), it is necessary to cover its orifice to achieve a proper landing zone. According to the literature, coverage of the LSCA was reported in nearly 50% to 84% of patients undergoing TEVAR in acute setting [4]. It is well known that the coverage of the left subclavian artery is associated with increased risk of stroke, spinal cord ischemia, and upper extremity ischemia [4,5]. Consequently, the Society for Vascular Surgery strongly recommends the revascularisation of the LSCA [3]. There are several endovascular options for proximal extension of the landing zone in the aortic arch with preservation of the blood flow in the LSCA. The chimney TEVAR with parallel implantation of a covered stent in the LSCA is a feasible alternative, but is connected with the potential risk of gutter endoleak [6,7]. Custom-made stent grafts are also available, but with long production time, and they are not appropriate to be implanted in acute settings [8]. The proposed off-the-shelf thoracic single side-branched stentgraft can be used only in a limited number of patients due to specific anatomic requirements [9]. In addition, no device has been approved by the regulatory authorities yet, and stentgrafts are mostly not available on the commercial market. Another interesting option is the in situ laser fenestration of the stent graft, although the broad application of this technique is limited due to the sophisticated equipment required and the increased risk of thermal damage to local tissue [10,11,12,13]. Due to these limitations, in recent years, a novel endovascular technique has evolved, named physician-modified TEVAR (pmTEVAR). In this operation, a commercially available aortic stentgraft will be modified by creating an appropriate fenestration for the LSCA directly prior to implantation. The purpose of our report was to compare the results of the pmTEVAR against hybrid repair of the proximal thoracic aorta in our centre.

## 2. Materials and Methods

### Study Design and Patient Selection

**Figure 1:**
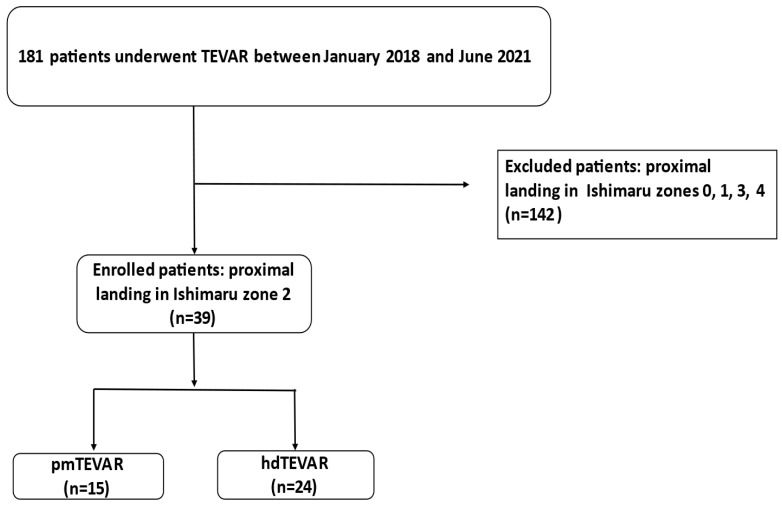
Flow diagram of inclusion process.

This was a single-centre, retrospective, observational study on a total of 181 consecutive patients who underwent TEVAR between January 2018 and June 2021, as the technique of physician-modified thoracic stentgrafts was introduced in our centre in January 2018 (Figure 1). Patient demographics, comorbidities, intraoperative data as well as pre- and postoperative computed tomographic angiogram (CTA) findings (maximum aortic diameter, landing zone), vital parameters and complications were analysed.

## 3. Exclusion Criteria

In our study, only patients with proximal landing zone of the stentgraft in Ishimaru zone 2 of the thoracic aorta were included. All patients who underwent a TEVAR with another proximal landing zone (zone 0: *n* = 16, zone 1: *n* = 1, zone 3 and 4: *n* = 125) were excluded from our study cohort (Figure 1). We used the following standard: Proximal stentgraft attachment site according to the Ishimaru classification: Zone 0: the proximal edge of the covered endograft is proximal to the innominate artery origin; Zone 1: distal to the innominate but proximal to the left common carotid artery (LCCA) origin; Zone 2: distal to the LCCA but proximal to the subclavian artery; Zone 3: 2 cm of the left subclavian artery without covering it; Zone 4: proximal extent of the endograft is 2 cm distal to the left subclavian artery and ends within the proximal half of the descending thoracic aorta (T6 approximating the midpoint of the descending thoracic aorta) [14]. To achieve an adequate proximal landing zone, the stent graft was deployed in Ishimaru zone 2 of the aorta, which led inevitably to the coverage of the LSCA. In all patients the patency of the LSCA was re-established. In the hybrid group pre or postinterventional debranching of the LSCA was undertaken, while in the pmTEVAR group a bridging stentgraft in the proximal LSCA was implanted.

Technical success was defined as successful stentgraft implantation in the thoracic aorta with revascularisation of the LSCA. Primary outcome was technical success, 30-day mortality, perioperative stroke, 30-day reinterventions and supraaortic access-related complications. Secondary outcomes were mortality, late complications, late reinterventions, and bypass/bridging stentgraft patency. The patients received a postprocedural contrast-enhanced computed tomography within 3 months postoperatively and thereafter annually.

## 4. The Operation Technique of pmTEVAR

**Figure 2:**
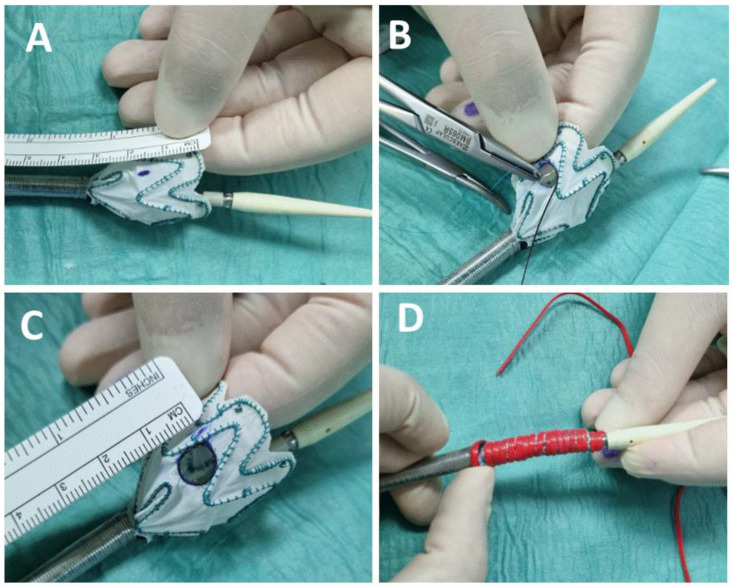
Stages of the operation technique: (**A**) partial deployment of the stentgraft; (**B**) creation of the fenestration and fixation of the radiopaque metal wire at the edge of the fenestration; (**C**) completion of the fenestration; (**D**) re-sheeting the stentgraft in the delivery system.

The technique was previously described [15]. In short:

The stentgrafts that were used in our study were Bolton Relay—NBS Plus or NBS Pro (Terumo Corp, Vascutek Ltd., Inchinnan, UK) and Zenith ZDEG, Cook Medical. The stentgraft was modified on a back table directly before implantation. The average duration of the stentgraft modification was 18 min. The stentgraft was partially deployed and a circular fenestration was marked on the stent graft in accordance with the preoperatively measured diameter of the LSCA. The fenestration was performed using a low-temperature handheld cautery device. A 0.018 wire was circular sown at the edge of the fenestration as a radiopaque marker for orientation under fluoroscopy. The stentgraft was wrapped with plastic vessel loops und re-sheeted in the delivery system (Figure 2). After an ultrasound-guided retrograde puncture of the common femoral artery two Preclose Pro Glide (Abbott Laboratories, Redwood city, CA, USA) devices were placed in the artery. Then, we performed an open surgical approach the left axillary artery and placed an arterial sheath. The stentgraft was then inserted over a stiff wire in the descending thoracic aorta through the femoral access. After adjusting the projection to the final angiography angle the stentgraft was oriented, so that the radiopaque marker faces the origin of the LSCA. Then we performed an aortography and carefully deployed the stentgraft under permissive hypotension or rapid pacing. After the graft deployment, the fenestration was retrograde cannulated through the left axilary artery and a bridging stentgraft was deployed. After removal of the arterial sheath the axillary artery was closed in a standard manner.

## 5. Statistical Analysis

Continuous variables are expressed as mean ± standard deviation for parametric data and median with interquartile range for non-parametric data, whereas dichotomous variables are presented as crude numbers and percentages. Comparisons of continuous variables were performed using Student’s *t* test for normally distributed variables, and a Mann–Whitney U test for non-normally distributed variables. A chi-square test or Fisher’s exact test was used for categorical variables. A *p*-value of <0.05 was considered statistically significant. All statistical analyses were performed using SPSS Statistics for Windows version 26.0 (IBM Corp., Armonk, NY, USA).

## 6. Results

During the study period of 42 months, 24 patients (59.9 ± 3.1 years; 19 male) were treated with a hybrid procedure (TEVAR with cervical debranching of the LSCA) and 15 patients (66.4 ± 3.05; 12 male) underwent pmTEVAR. The baseline characteristics of the patients are given in Table 1. No significant differences in terms of comorbidities in were detected in either group.

A total of 67% of the patients (*n* = 16) of the hdTEVAR group and 67% (*n* = 10) from the pmTEVAR group were operated on due to type B aortic dissection. Of those, 69% (*n* = 11) vs. 20% (*n* = 2), respectively, underwent treatment in acute settings, while 31% (*n* = 5) vs. 80% (*n* = 8) were operated electively. For the rest of the patients: 33% (*n* = 8) of the hybrid TEVAR group vs. 33% (*n* = 5) of the pmTEVAR underwent the operative treatment because of a complex TAA or TAAA. Of those, 25% (*n* = 2) vs. 60% (*n* = 3), respectively, were operated in an acute setting, and 75% (*n* = 6) vs. 40% (*n* = 2), respectively, underwent an elective surgical procedure. The mean follow up was in the hdTEVAR Group 17.4 ± 2.5 months and 10.4 ± 2.3 months in the pmTEVAR.

## 7. Intraoperative Findings

The median operation time was 188 min (169–238) in the pmTEVAR group and 279 min (211–334) in the hdTEVAR group, (*p* = 0.003). The postoperative observation at the intensive care unit was on average 6 days for the hybrid TEVAR group, compared to the significantly lower rate of 2.5 days for the pmTEVAR patients, (*p* = 0.022). The In-hospital-length of stay was also significantly longer for the TEVAR with cervical debranching group: 14.5 vs. 7 days, respectively (*p* = 0.001). Technical success was achieved in 100% of the patients from both groups. No significant difference was revealed with respect to the amount of the contrast medium or the intraoperative fluoroscopic time, or with respect to the average length of the implanted stentgraft: 199 mm vs. 154 mm in the hybrid TEVAR and pmTEVAR, respectively (*p* = 0.319). The average proximal stentgraft diameter in both groups was 36 mm, while the distal diameter in the hdTEVAR group was 34 mm vs. 36 mm in the pmTEVAR group, *p* = 0.837, Table 2.

## 8. Mortality

The 30-day mortality was 12.5% (*n* = 3) in the hdTEVAR group vs. 6.66 % (*n* = 1) in the pmTEVAR cohort (*p* = 0498). One of the patients from the hdTEVAR group, who underwent TEVAR, bEVAR, EVAR and carotid-subclavian bypass due to symptomatic Type II TAAA, died on the 18th postoperative day due to diffuse bilateral ischemic stroke after successful cardiopulmonary resuscitation because of acute bradycardic syndrome. The second patient died on the 8th postoperative day due to major stroke after supracoronary ascendens and aortic arch replacement after postoperative retrograde type A aortic dissection. The indication for index procedure was an acute complicated TBAD with visceral malperfusion. The third patient died due to haemorrhagic shock and multiorgan dysfunction syndrome as a consequence of a ruptured type II TAAA. In the group of pmTEVAR, one patient died due to tetraplegia and pulmonary insufficiency caused by intraspinal bleeding from a cerebrospinal fluid drainage. The indication for the operative treatment was a progressive dilatation in the acute phase of intramural haematoma/aortic dissection with progressive ulcer like projections. The patient was treated years before with EVAR and some months before with TEVAR and bEVAR. In the follow-up, no patients died.

## 9. Perioperative Stroke

Major strokes occurred in two hybrid TEVAR patients (8.3%), both with lethal consequences. One stroke occurred after open ascendens and arch replacement and was not correlated with the index procedure. The second patient suffered a diffuse hemodynamic bilateral ischemic brain damage due to a prolonged but eventually successful cardiopulmonary resuscitation due to acute bradycardic syndrome. In the pmTEVAR group, no patients suffered minor or major stroke (*p* = 0.54), Table 3.

## 10. Access-Related Complications

In the group of hdTEVAR, two patients (8.3%) suffered a minor postoperative neck haematoma, both of which were treated conservatively. Only one of the pmTEVAR patients developed a postinterventional groin haematoma, which also resolved without operative treatment. We failed to detect any clinical or radiological signs of nerve injuries in our hdTEVAR cohort. One patient of the pmTEVAR group suffered acute hand ischaemia due to a postoperative occlusion of the brachial artery as a result of intraoperative arterial dissection. He underwent an emergent thrombectomy with patchplasty of the brachial artery, Table 3.

## 11. Reintervention

A total of 8.3% of the patients with TEVAR and cervical debranching (*n* = 2) vs. 6.66% from the pmTEVAR patients (*n* = 1) underwent a reintervention within the first 30 days after the operation. In one of the patients from the hybrid TEVAR group, a bypass thrombectomy was performed on the first postoperative day due to acute bypass occlusion. Another patient from the same group underwent an emergent replacement of the ascendent thoracic aorta and the aortic arch due to a postimplantation retrograde type A aortic dissection. In the group of the pmTEVAR only one patient from the study cohort underwent a reintervention within the first 30 postoperative days. In this case, we performed at the same day of the index procedure a thrombectomy with patchplasty of the left brachial artery due to acute arterial occlusion as a consequence of a local arterial dissection. In the mid-term follow up no reinterventions were performed due to complications of the carotid-subclavian bypass, whereas one patient from the pmTEVAR group underwent a late reintervention. In this case, we performed distal extension of the bridging stentgraft of the LSCA due to an endoleak type Ic. The secondary patency rate of the bypass grafts and the primary patency of the bridging stents in the LSCA was 100%.

## 12. Discussion

It is well known that the endovascular treatment of aortic lesions, located in the proximal descending thoracic aorta, often requires coverage of the LSCA in order to achieve an appropriate proximal landing zone with secure und adequate stent graft fixation. A significant number of studies have clearly demonstrated, that the LSCA is an important source of perfusion of the brain, spinal cord, and the left upper extremity [4,5]. This, in turn, as a consequence of the intentional LSCA coverage without a previous revascularisation, increases significantly the risk of postoperative stroke, spinal cord ischemia, or ischemia of the left upper extremity [16,17]. In patients with a previous coronary revascularisation through the left internal mammary artery, the coverage of the LSCA would result in a myocardial ischemia with potential lethal consequences. The purpose of our study was to compare the outcome of the recently developed technique of physician-modified TEVAR with the standard hybrid operation in terms of TEVAR combined with a cervical debranching in means of carotid-subclavian bypass. For the hybrid operation we used predominantly a standard supraclavicular approach to the common carotid und subclavial artery. Rarely, we performed the carotid-subclavian bypass through two separate supra- and infraclavicular arterial approaches. In accordance with the arterial diameter, a 6 mm or 8 mm polyester graft was used. According to the literature, the most common perioperative complication of the hybrid operation is the nerve injury (N. phrenicus-25%, N. laryngeus recurrens-5%, N. axillaris-2%) [18]. In our study, in patients who underwent a carotid-subclavian bypass operation, a nerve injury in the postoperative period could not be detected. Two patients of the hybrid group (8.3%) suffered a postoperative stroke, while in the group of the pmTEVAR, none of the patients had a brain ischemia. Unfortunately, both patients died in the early postoperative period exactly due to this complication. In our cohort, only one patient underwent a reoperation due to acute bypass occlusion on the first postoperative day. According to the bypass and stent graft patency, no significant difference was detected in either group. The secondary bypass patency and the primary bridging stent graft patency during the midterm follow up period in all patients was 100%. As Table 3 demonstrates, there is a statistically significant difference in terms of postoperative ICU-stay and the In-hospital-length of stay between both groups. The patients operated with pmTEVAR had a mean ICU-Stay of 2.5 days, compared to 6 days in the hybrid operation group, which is more than 50% less. The mean in-hospital-length of stay of the hybrid group patients was 14.5 days vs. 7 days for the pmTEVAR-patients. However, we must take into consideration that most of the emergent operated patients with less time for proper preoperative preparation and accordingly higher risk of postoperative complications were from the hdTEVAR group, 54% (*n* = 13) vs. 33% (*n* = 5).

It is also of note that the duration of the pmTEVAR operation was, on average, 82 min shorter, compared to the hybrid operation which is not to underestimate especially by polymorbid and high-risk patients. The literature review reveals that this issue is supported by another authors [7].

Both the entirely endovascular pmTEVAR and the hybrid TEVAR with debranching offer a safe and effective perfusion of the left subclavian artery by treatment of proximal lesions of the thoracic descendent aorta with 100% bypass and LSCA- bridging stent patency rate in the midterm follow up period. Our study demonstrated that the rate of the procedure related complications in terms of periprocedural strokes, extremity ischaemia, access complications, as well as the early postoperative reinterventions in both groups are comparable. However, for the patients from the pmTEVAR group, a shorter operation time (188 vs. 279 min, *p* = 0.003), as well as ICU observation time (2.5 vs. 6 days, *p* = 0.22) and total in-hospital length of stay (7 vs. 14.5 days, *p* = 0.001) by approximately equivalent amount of contrast medium and fluoroscopic time in both groups was detected.

## 13. Limitations

Among the limitations of the current study should be mentioned its single-centre retrospective character, as well as its small sample size. Furthermore, the short follow up period may have influenced the occurrence of the long-term complications or the bypass and LSCA-stent patency.

## 14. Conclusions

Based on our study, we concluded that the patency of the left subclavian artery in the setting of TEVAR with proximal landing in Ishimaru zone 2 can be safely and effectively maintained with both above mentioned hybrid and complete endovascular surgical techniques. However, the pmTEVAR appears to have some advantages compared to the TEVAR with debranching in terms of potentially lower postoperative complication rate, as well as a tendency to lower mortality rates. Certainly, a long-term follow up is necessary to determine the rate of late complications in terms of stroke, bypass/LSCA-bridging stent patency rate and endoleak.

## Figures and Tables

**Table 1:** Baseline characteristics of the study population.

Variables	Total (*n* = 39)	HdTEVAR (*n* = 24)	PmTEVAR (*n* = 15)	*p*-Value
**Demographic characteristics**				
Age (years), mean ± SD	62.2 ± 2.5	59.6 ± 3.7	66.4 ± 3.05	0.383
Male gender, *n* (%)	31 (79.4)	19 (79.1)	12 (80)	0.640
BMI kg/m^2^, mean ± SD	28 ± 1	29 ± 1.4	26.1 ± 0.9	0.289
**Type of aortic pathology, *n* (%)**				
Dissection	27 (69.2)	16 (66.6)	10 (66.6)	
Aneurysm	12 (30.7)	8 (33.3)	5 (33.3)	
Max. diameter (mm), median (IQR)	74.5 (61.5–86.7)	61 (60–61)	73.5 (64–106.2)	0.133
**Medical history, *n* (%)**				
Hypertension	35 (89.7)	21 (87.5)	14 (93.3)	0.498
Previous stroke/TIA	5 (12.8)	4 (16.6)	1 (6.6)	0.351
COPD	7 (17.9)	4 (16.6)	3 (20)	0.556
Diabetes	4 (10.2)	3 (12.5)	1 (6.6)	0.498
Coronary artery disease	3 (7,6)	1 (4.1)	2 (13.3)	0.326
Chronic heart disease	7 (17.9)	5 (20.8)	2 (13.3)	0.444
Current/previous smoker	9 (23)	4 (16.6)	5 (33.3)	0.061
Atrial fibrillation	6 (15.3)	4 (16.6)	2 (13.3)	0.579
**Medication treatment, *n* (%)**				
ß-Blocker	31 (79.4)	20 (83.3)	11 (73.3)	0.261
ACEs-Inhibitor	13 (33.3)	9 (37.5)	4 (26.6)	0.332
Aspirin	27 (69.2)	14 (58.3)	13 (86.6)	0.087
Statin	18 (46.1)	9 (37.5)	9 (60)	0.177

SD, standard deviation BMI, body mass index; IQR, interquartile range; TIA, transient ischemic attack; COPD, chronic obstructive pulmonary disease ACEIs, angiotensin-converting enzyme inhibitors.

**Table 2:** Procedural data.

Variables	Total (*n* = 39)	HdTEVAR (*n* = 24)	PmTEVAR (*n* = 15)	*p*-Value
**Procedure characteristics**				
OP duration (min), median (IQR)	235 (188–310)	279 (211–334)	188 (169–238)	**0.003**
ICU stay (days), median (range)	4 (1–23)	6 (2–23)	2.5 (1–13)	**0.022**
In-hospital length of stay (days), median (range)	11 (4–118)	14.5 (4–79)	7 (5–118)	**0.001**
Contrast medium, mean ± SD	85.07 ± 6.2	92.9 ± 8.9	75.6 ± 7.3	0.248
Fluoroscopic time, mean ± SD	39.4 ± 4.9	36.7 ± 7.7	42.6 ± 5.8	0.145
Length of stent graft, median (range)	164 (114–259)	199 (114–259)	154 (145–250)	0.319
prox. diameter, median (range)	36 (28–46)	36 (28–46)	36 (30–46)	0.607
dist. diameter, median (range)	35 (28–46)	34 (26–46)	36 (30–46)	0.837

ICU, intensive care unit; Statistically significant *p*-values are marked in bold.

**Table 3:** Thirty-day complications rate and procedure related adverse events.

Variables	Total (*n* = 39)	HdTEVAR (*n* =24)	PmTEVAR (*n* = 15)	*p*-Value
Thirty-day complications rates, *n* (%)				
Death	4 (10.2)	3 (12.5)	1 (6.66)	0.498
Stroke	2 (5.1)	2 (8.3)	0 (0)	0.372
Access complication	4 (10.25)	2 (8.3)	2 (13.3)	0.673
Reintervention	3(7.7)	2 (8,3)	1 (6.66)	0.736
Discharge home	34 (87.1)	20 (83.3)	14 (93.3)	0.351
Renal failure	1 (2.5)	2 (8.3)	0 (0)	0.615

## Data Availability

Not applicable.
